# 

**Published:** 1898-07

**Authors:** 


### BOOKS RECEIVED.

Hay Fever and Its Successful Management. By W. C. Hollopeter, A. M., M. D., Clinical Professor of Pediatrics in the Medico-Chirurgical College of Philadelphia; Physician to the Methodist-Episcopal Hospital, etc., etc. Duodecimo, pp. 137. Price, $1.00, net. Philadelphia: P. Blakiston, Son & Co. 1898.

Atlas and Abstract of the Diseases of the Larynx. By Dr. L. Grünwald, of Munich. Authorised translation from the German. Edited by Charles P. Grayson, M. D., Lecturer on Laryngology in the University of Pennsylvania, etc., etc. With 107 colored figures and forty-four plates. Price, $2.50, net. Philadelphia: W. B. Saunders. 1898.

Modern Gynecology. A Treatise on Diseases of Women. Comprising the results of the latest investigations and treatment in this branch of medical science. By Charles H. Bushong, M. D., Assistant Gynecologist and Pathologist to the Demilt Dispensary, New York; formerly Attending Physician to the Northern Dispensary, New York. Illustrated. Second edition, enlarged. New York: E. B. Treat & Co. 1898.

Twentieth Century Practice. An International Encyclopedia of Modern Medical Science. By leading authorities of Europe and America. Edited by Thomas L. Stedman, M. D., New York City. In twenty volumes. Volume XIV., Infectious Diseases. New York: William Wood & Co. 1898.

Atlas of Legal Medicine. By Dr. E. v. Hofmann, Professor of Legal Medicine and Director of the Medico-Legal Institute at Vienna. Authorised translation from the German. Edited by Frederic Peterson, M. D., assisted by Aloysius O. J. Kelly, M. D. Fifty-six plates in colors and 193 illustrations in black. Price, $3.10 net. Philadelphia: W. B. Saunders. 1898.

Medical and Surgical Report of the Presbyterian Hospital in the City of New York. Edited by Andrew J. McCosh, M. D., and Walter B. James, M.D. Volume III. January, 1898.

Diseases of the Stomach. A Text-book for Practitioners and Students. By Max Einhorn, M. D., Instructor in Clinical Medicine at the New York Post-Graduate Medical School and Hospital; Visiting Physician to the German Dispensary. Second revised edition. 502 pages. Price, cloth, $3.25 net; flexible leather, $3.75 net. New York: William Wood & Co. 1898.

Diseases of the Skin. Their Constitutional Nature and Cure. By J. Compton Burnett, M. D. Third edition, revised and enlarged. Price, cloth, by mail, $1.07 net. Philadelphia: Boericke & Tafel. 1898.

Yellow Fever. Clinical Notes. By Just Tonatre, M. D. (Paris), Member Board of Experts Louisiana State Board of Health. Translated from the French by Charles Chassaignac, M. D., President New Orleans Polyclinic; Editor New Orleans Medical and Surgical Journal. New Orleans: New Orleans Medical and Surgical Journal, Limited. 1898.

---

### ITEMS.

### Index to Volume XXXVII.

### AUGUST, 1897—JULY, 1898.

PAGE.

ABORTION, Curettage after . . 7
Acetonuria . . . . . . . . 430
Adirondacks in Winter for Tubercular Patients . . . . . . . . 657
Advertisement in the Herald . . . 932
Alabama System . . . . . . . . 607
Albuminuria, Functional . . . . . 769
of Pregnancy . . . . 258
Alimentary Canal, Nutrose in Diseases of . . . . . . . . . . . 271
Alpha Naphthol, Uses of . . . . . 368
Anemia Following Diarrhea . . . 426
Significance of Chlorides in . . . . . . . . 365
Treatment of . . . . . . 89
Angell, Edward B., Hygiene of the Mind . . . . . . . . . . . 241
Antitoxic Serums . . . . . . . . 881
Antitoxin, Administration and Value of . . . . . . . . . . . . . . . 97
Appendicitis and Intestinal Obstruction, Differential Diagnosis of . . . . . . . 530
Relation of to Uterine Adnexæ and Pregnancy . . . . . . . . . 248
with Unusual Sequelæ . 733
Army and Navy Notes . . 785, 856, 938
Arsenauro, Therapeutic Value of . 608
Aseptic Method in Surgery, Evolution of . . . . . . . . . . . . 920

BARBER Shop as a Menace to Public Health . . . . . . 801
Becker, Tracy C., Expert Testimony . . . . . . . . . . . . . 561
Blepharitis, Treatment of . . . . 767
Blood, Changes of Fat of Chyle in, 366
Chlorides and Phosphates . 366
Blood-letting as a Therapeutic Measure . . . . . . . . . . 192
Blume, F., Dynamic Ileus Following Operations . . . . . . . . 161
Books Received . 73, 155, 238, 316, 395, 475, 557, 637, 715, 797, 876, 943
Breast-milk, Chemical Examination of . . . . . . . . . . . . . . . 39

PAGE.

Bronchial Catarrh . . . . . . . . . 113
Pneumonia . . . . . . 112
Bronchus, Foreign Body in . . . . 19
Brown, J. C., A Case of Corrosive Poisoning . . . . . . . . . . . 103
Burns, Treatment of . . . . . . . 680

CARBOHYDRATE, Transformation of Fat into . . . . . 427
Carcinoma in the Negro . . . . . 174
Carpenter, Thos. B., Genito-urinary Epithelium . . . . . . . . . . . 651
Cesarean Section, Post Mortem . . 512
Children, Dependent . . . . . . . 454
Chloroform Collapse, Symptoms of, 127
Choline, Physiological Action of . . 367
Christian Science, Death of Child Treated by . . 296
Editor of the Buffalo Enquirer . 538
Healers, Arrest of . . . . . . 456
Christian, The, by Hall Caine . . . 299
Circumcision . . . . . . . . . . . 677
Clemesha, J. C., Thomsen's Disease, 16
Colleges, Three Years . . . . . . 284
Concretions, Multiple Intestinal . . 366
Conjunctiva, Purse String Suture of, 688
Corneal Ulcers, Galvanic Current in . . . . . . . . . . . . . . 25

**Correspondence:**

Carcinoma in the Negro, J. Collins Warren . . . . . . . . . 369

Cott, Geo. F., Administration and Value of Antitoxin . . . . . . 97
Cough Mixture . . . . . . . . . . 914
Creveling, J. P., Surgical Treatment of Tubercular Peritonitis . . . . 409
Crime and Degenerates . . . . . 456
Crockett, Montgomery A., The Philosophic Spirit in Science . . . . 321
Cronyn, John, In Memoriam . . . . 591
Cumston, Charles Greene, Gangrene due to Arterio-sclerosis . . . . . 721
Curettage after Abortion . . . . . 7

PAGE.

DACRYOCYSTITIS, Etiology of . . . . . . . . . . . . . . 431
Daggett, B. H., Gonarthritis . . . 588
Circumcision . . . 677
Davis, Richard Harding, and the Surgeons . . . . . . . . . . . . 933
Deaver, John B., Appendicitis in Disease of Uterine Adnexæ and Pregnancy . . . . . . . 248
Dengue Fever, Epidemic of in Asia Minor . . . . . . . . . . 375
Dennis, John, Fluorometer in X-ray Work . . . . . . . . . . . 666
Dental Examiners, New York State, 283
Dentistry, Illegal Practice of . . . 375
Diabetes, Phloridzin . . . . . . . 365
Diarrhea, Summer . . . . . . . . . 919
Diarrheal Diseases of Children . . 581
Diet, Value of Change of . . . . 38
Digestion, Salivary . . . . . . . 11
Diphtheria . . . . . . . . . . . 684
Dowd, J. Henry, Chronic Specific Urethritis . . 1
Gonorrhea in Women . . . 338
Stricture of the Urethra . . . 908
Dropsies, Obstinate . . . . . . . 845
Dysmenorrhea . . . . . . . . . 590

ECTOPIC Pregnancy . . . . . 438
Eczema, Picric Acid in . . . 272
Elsner, S. L., Case of Puerperal Diphtheria . . . . . . . . . . 842
Enteroptosis . . . . . . . . . . 680
Epilepsy, A Case of Jacksonian 663
Factors in Production of, 822
Examination, Entrance at Univ. of Pa . . . . . . . . . . . . . . 115
Examinations, Gems from . . . . 279
Examining Board, First N. Y. State Medical . . . . . . . . . . . . 445
Expert Medical Testimony . . . . 417
Medical Testimony, Legislation in Regard to . . . 492
Testimony . . . . . . . . 332
Testimony from a Legal Standpoint . . . . . . 561
Eucaine Anesthesia . . . . . . . 431
Eye, Gunshot Wound of . . . . . 28
Operations, Painless . . . 688
Method of Preserving for Demonstration . . . . . . 767

FACE Presentations . . . . . . 42
Fluorometer in X-ray Work . 666
Frederick, C. C., When Shall the Uterus be Removed in Surgical Work? . . . . . . . . . . . . . 750

PAGE.

Fronczak, Francis E., Cerebral Meningitis . . . . . . 345
Case of Post Mortem Cesarean Section . 512

GANGRENE due to Arteriosclerosis . . . . . . . . 721
Garbage Contract . . . . . . . . . 854
Genito-urinary Epithelium . . . . 651
Glaucoma, Iridectomy in . . . . 356
Goldberg, S., Curettage after Abortion . . . . . . . . . . . . 7
Gonarthritis . . . . . . . . . 433, 588
Gonorrhea in Women . . . 338
Neisser's Method of Treating . . . 768
Permanganate of Potassium in . . . . . 436
Silver Salts in . . 34
Guillemont, F., Pathology and Treatment of Locomotor Ataxia . 904

HAIR, To Remove Superfluous, 682
Hammond, Charles Willard, Memorial of . . . . . . . . 263
Handkerchiefs, Promiscuous Use of . . . . . . . . . . . . . . . 292
Harelip, Complicated Double . . . 401
Hay Fever, Treatment of . . . . 288
Hematoma, Orbito-palpebral . . . 29
Hemianopia, Transient Varieties of, 765
Henckell, A. W., Expert Medical Testimony . . . . . . . . . . 417
Himmelsbach, George A., Foreign Body in Bronchus . . . . . . . 19
Hospitalism in Infants . . . . . 36
Houghton, E. M., Antitoxic Serums. 881
House, William, Factors in Production of Epilepsy . . . . . . . . 822
Howe, Lucien, Purulent Ophthalmia . . . . . . . . . . . . 585
Hydrocephalus, Three Cases of . . 259
Hydrozone in Disorders of the Genito-urinary Tract . . . . . 362

IDAHO State Board of Medical Examiners . . . . . . . . . . 118
Ileus, Dynamic after Operation . . 161
Illinois, Medical Registration in . 286
Nongraduate Applicants in, 601

**Illustrations:**

Broncho-pneumonia (Williams) 745
Cronyn, John . . . . . 621
Defective Development in an Infant (Roe). Figure 1. Child Five Weeks Old before Operation . . . . . . . . . 402

PAGE.

Figure 2. Cleft Through Hard and Soft Palate . . . 403
Figure 3. Closure of Maxillary Fissure . . . . . . . . 404
Figure 4. Child Ten Months Old after Operation . . . . 405
Figure 5. Child Laughing, Showing Contraction of Lip, 406
Epithelioma of the Esophagus (Williams) . . . . . . . . . 746
First State Medical Examining Board—William C Wey, William S. Ely, Eugene Beach, Joseph P. Creveling, George Ryerson Fowler, Maurice J. Lewi, William Warren Potter, facing . . . 445
Flint, Austin . . . . . . . . 763
Gay, Charles C. F . . . . . . 840
Hamilton, Frank Hastings . . 832
Hunt, Sanford B . . . . . . 837
Instrument to Determine Visual and Color Fields(Ringueberg) 511
Javal Ophthalmometer (Satterlee) . . . . . . . . . . . 774
Multiple Idiopathic Pigmented Sarcoma (Wende) . . . . . 820
Rochester, Thomas F . . . . 839
Smith, J. Greig, facing . . . 401
Storck, Edward . . . . . . 841
Tubercle Bacilli in Sputum (Orr) . . . . . . . . . . . 748
View of Buffalo, 1845 . . . . 830
Wey, William C . . . . . . . 55
White, James Platt . . . . . 760

Infants, Difficult Defecation in . . 686
Infection, Prenatal . . . . . . 425
Influenza, Calomel to Abort . . 681
Insomnia, Treatment of . . . 533, 682

**Instruments, New:**

Instrument to Determine Visual and Color Fields . . . . 511
Satterlee's Model of the Javal Ophthalmometer . . . . . 774

Intestinal Catarrh, Treatment of . 533
Obstruction, Operation for . . . . . . . . . . . . 426
Intestine, Resection of Gangrenous, 514
Intussusception Following Dysentery . . . . . . . . . . . . . . 685
Iowa Medical Practice Law . . . 118
Iron, Therapeutics of . . . . . . 108

**Items:**

American Medical Association Railway Rates . . 880
Pediatric Society, 240,479

PAGE.

American Public Health Association . . . . . 400
Annual Report Buffalo Children's Hospital . . 559
Report Maryland, Second, Hospital for the Insane . . . . . . 559
Report Sheppard Asylum . . . . . . . . 558
Antikamnia Chemical Co . . . 400
Antipyrin, Reduction in Price of . . . . . . . . . . . 879
Army Medical Board . . . 78, 639
Attack on Vivisection . . . . 77
Banquet, Clinical Society of Louisville . . . . . . . . . . 79
Beef Juice . . . . . . 400
Chutmuck Special . . 559, 720, 880
Clyde Line Fleet . . . 560, 719
Delegation of Mexican Physicians . . . . . . . . . . . . 79
Effervescent Lithia Tablets . . 80
Fined Under Medical Registration Law . . . . . . . . . 479
Flavell, G. W. & Bro . . . . . 944
Friable Pills . . . . . . 479
Governor Tanner and Osteopathy . . . . . . . . . 79
Grand Army of the Republic . 78
History of Medicine County of Erie . . . . . . . . . . . 639
International Health Exposition . . . . . . . . 560
Journal Advertising Agency . 800
M. J Breitenbach Company . 718
Mathews's Quarterly Journal Special Train . . . . . . . 559
Medical Excursion to Salt Lake City . . . . . . . 640, 800, 879
Medical Society County of Erie, Counsel for . . . . . 640
Medical Society of London, Engraving of . . . . . . . 879
Mellin's Food Company . . . 944
Midwinter Sun Baths . 480
National Association for the Study of Epilepsy . . . . 718
New York Board of Trade and Transportation . . . . . . 720
Orthoform . . . . . . . . . 640
in Military and Naval Surgery . 879
Stockwell, Dr. G. A . . . 559
Sweetwater Hotel, Bedford Springs, Mass . . . . . . . 80
Taka-diastase . . . . . . . . 943
University Club . . . . . 719
Victor Koechl & Co., Removal of . . . . . . . . . . . . . 800
Wampole's War Atlas . . . . 944

PAGE.
Wyeth's Phosphate of Sodium, 718
Wyeth, John & Bro . . . . . 944

JACOBSON, Nathan, Appendicitis with Unusual Sequelæ . . . 733
Jew, The, in Medicine . . . . . 481
Jones, Frank A., Resection of Gangrenous Intestine . . . . . . . 514
Journalism, Courtesies of . . . . 373

KANSAS Needs a Practice Law . . . . . . . . . . 604
Kidney, Tabes and Movable . . . 271
Kiepe, Edward J., Salivary Digestion . . . . . . . . . . . . 11
Kindergarten Work and Eyesight . 354
Kinnear, Beverly O., Treatment of Anemia . . . . . . . . . . 89
Klondike Doctors Must Pass Examining Board . . . . . . . . 848
Krauss, William C., Nitroglycerin in Trigeminal Neuralgia . . . . 169

LACHRYMAL Duct, Obstruction of . . . . . . . . . . . . 42
Lachrymal Obstructions . . . . . 27
Laboratory Methods in Medicine . 745
Larrey, Baron, Anecdote of . . . 853

### Leading Articles :

American Association of Obstetricians and Gynecologists . 132
And War Came . . . . . . . 781
Autumn Society Meetings . . 226
Buffalo Pathologic Library . . 852
Consolidation of Buffalo Medical Schools . . . . . . . . 931
Free Public Baths . . . . . . 51
G. A. R. Hospital Service . . 135
In Case of War . . . . . . 692
Kentucky's Triumph . . . . . 134
Medical Legislation at Albany, 694
Society County of Erie and the District Attorney's Office . . . 535
Society State of New York . . . . . . . 612
Staff of the Army . . 929
Reform in Expert Medical Testimony . . . . . . . . . . . 293
Roof Gardens for the People . 53
Shock . . . . . . . . . . . 615
State Care of Consumptives . 616
The Mayor-elect . . . . . . . 370
University of Buffalo, Annual Commencement . 775
of Niagara, Annual Commencement . 849

PAGE.
Year of Medicine in Buffalo . 450
Yellow Fever in the South . . 229

License to Practise, The State . . 669
Lichty, J. A., Uses of Stomach Tube in General Practice . . . . . . 896
Life Insurance, Gonorrheic Applicants for . . . . . . . . 265
Litholapaxy Under Local Anesthesia . . . . . . . . . . 129
Liver, Coagulating Properties of . 427

### Literary Notes :

Alabama Medical and Surgical Age . . . . . . . . . . . 239
Albany Medical Annals . . . 478
American Journal of Insanity . 878
Monthly Microscopical Journal . . . 558
X-ray Journal . . . 75
Annual Report St. Lawrence State Hospital . . . . . . . 156
Arsenauro and Mercauro . . . 800
Brigham Hall . . . . . . . . . 878
Buffalo General Hospital, Annual Report . . . . 878
Medical Journal . . . 798
State Hospital Report, 74
Bulletin, Harvard Alumni Association . . . . . . . . . . 318
Canadian Journal of Medicine and Surgery . . . 240
Practitioner . . . . 240
Chicago Clinic . . . . . . . . 399
Cincinnati Hospital Report . . 157
Clyde Line . . . . . . . . . . 639
Columbia Calendar (1898) . . 399
Columbus Medical Journal . . 77
Current Thought . . . . . . . 477
Dominion Medical Monthly . 240
Edwards's Journal of Health . 319
Georgia Journal of Medicine and Surgery . . . 156, 477
Indiana Medical Journal . 75
Iris, The . . . . . . . . . 798
International Journal of Surgery . . . . . 798
Medical Annual . 558
Medical Magazine . . . 478, 876
Laryngoscope, The . . . 477, 877
Lea Brothers & Co., Announcement . . . . . . . . . . . 799
Lofoten Islands . . . . . . . 638
Louisville Journal of Medicine and Surgery . . . . . . . . 717
Manhattan Eye and Ear Hospital Report, Volume IV . . 74
Massachusetts State Board of Health . . . . . . . . . . 878

PAGE

Medical Age . . . . . . . 558
Council . . . . . . . 318
Mirror . . . . . . . . 717
News Visiting List (1898) . . . . . 398
Record . . . . . . . 74
Register . . . . . . 75
Memphis Lancet . . . . . . 877
Montgomery A. Crockett on Medical Teaching . . . 240
Monthly Cyclopedia of Practical Medicine . . . . . . . 639
National Hospital and Sanitarium Record . . . . 319
Medical Review . 318, 877
Needs and Rights of Old Age, 156
New Hampshire Sanitary Journal . . . . . . . . . . . 876
New Orleans Medical and Surgical Journal . . 74
North Carolina Medical Journal . . . . . . . . . . 717
Northern Druggist . . . . . 77
Pennsylvania Medical Journal, 77
Philadelphia Medical Journal . . . . . . . 398, 557
Polk's Medical and Surgical Register . . . . . . . 718, 798
Practical Therapeutics . 717
Report of Ohio State Board of Medical Registration and Examination . . . . . 318
Sajous's Annual of Practical Medicine . . . . . . . 877
Saunders's Book List . . . . 76
Second Hospital for the Insane of Maryland Report . . . . 75
Texas Medical News . . . . . 717
Therapeutic Progress . . . . 477
W. B Saunders's Announcement . . . . . . . . . 557
Western Medical and Surgical Gazette . . . . . 239
William Wood & Co., Announcement . . . . . . 240
Wisconsin Medical Recorder . 717

Locomotor Ataxia, Pathology and Treatment . . . . . . . . . . 904
Longevity and the Brain . . . . . 457
Loveland, B. C., Errors in Treatment of Nervous Prostration . . 738

MAINE, United States Battle-ship, Disaster to . . . . . . 618
Malaria, Action of Quinine in . . . 111
Forty-nine Cases of . . . 270
Mammary Fibro-cystic Degeneration . . . . . . . . . . . . 69
Manila Bay, Battle of . . . . . . 854

PAGE.

Maryland, Illegal Practitioners in . 285
Medical Examining Board . . . . . . . . . 600
Maturity, Precocious . . . . . . 691
Medical Board, Right of Appeal from . . . . . . . . . . . . . 114

### Medical College, Hospital and Dispensary Notes:

Bellevue Hospital Medical College . . . . . . . . . . 383
Brooks Memorial Hospital, Dunkirk . . . . . . . . 862
Buffalo German Hospital . 67, 548
Hospital Sisters of Charity . . . . . 304
Polyclinical Association . . . . . 628
University Medical College, 231, 305, 384, 702
Cornell University Hospital . . . . . . . . . . . . . . 464, 788
Erie County Hospital staff . . 465
Hospital for Consumptives . . 68
Jennie Casseday Free Infirmary for Women . . . . 705
Manhattan Eye and Ear Hospital . . . . . . . . . . 705
Massachusetts Hospital for Consumptives . . . . 464
Medico-Chirurgical College of Philadelphia . . . . 862
Memphis Hospital Medical College . . . . . . . . 465
New York Post-graduate Medical School and Dr. Kelsey . . . 863
Skin and Cancer Hospital . . . . 703
Niagara University Medical College . 231, 304, 702, 788
Niagara and Buffalo, Union of. 937
North Tonawanda Hospital . . 548
Philadelphia Polyclinic . . 305, 789
Pittsburg Hospital for Women, 862
Riverside Hospital . . . . . . 862
Rush Medical College . . . . 549
University and Bellevue Hospital Medical College . . . . 861
of Cincinnati . . . 68
Woman's Hospital, New York . 463

Medical Education, Center of . . 603
History of the County of Erie . . . . 641, 754, 829
Practitioner, Punishment of . . . . . . . . . . 848
Record, *Faux Pas* of . . . 696

PAGE.

Meningitis, Three Cases of Cerebral . . . . . . . . 345
Menstruation, Influence of Climate on . . . . . . . . . . . . . 42
Menthol for Insect Bites . . . . . 424
Metabolism, Muscular Power and Gaseous . . . . . . . . . . . . . 364
Milk, Value of Sterilised . . . . . 439
Mind, Hygiene of . . . . . . . . 241

**Miscellany:**

Adulteration of Foods and Drugs . . . . . . . . . . . 319
Second-hand Corks . . . . . . 160
Shakespeare Revised . . . . . 320
Study of American Medicinal Flora . . . . . . . . . . . . 157

Murray, C. A., Bloodletting as a Therapeutic Measure . . . . . . 192

NEPHRITIS, Analysis of Urine in . . . . . . . . . . 357
Clinical Classification of . . . . . . . . . 267
Diagnosis of Chronic . 122
General Management of Chronic . . . . . . . . 120
General Pathology of . 360
Interstitial and Parenchymatous Differentiated . . . . . . . . 31
Prognosis of Chronic . 121
Treatment of Chronic . 119

Nerve Element in Surgical Pathology . . . . . . . . . 125
Nervous Prostration, Errors in Treatment of . . . . . . . . . . 738
Neuralgia, Treatment of Trigeminal . . . . . . . . . . . . . . 169
Neuritis, Retroöcular . . . . . . . 26
Newspaper Medical Literature . . 932
Noises, Street . . . . . . . . 443
and Nuisances, Street . . . 783

**Obituary:**

Bates, Newton L. . . . . . . 301
Bradley, Alfred E. . . . . . 699
Brown, Bedford . . . . . . . 302
Cronyn, John . . . . . . . . . 619
Cullinane, John J. . . . . . . 784
Gibbs, John Blair . . . . . . 934
Hamlin, Charles Willard . . . 301
Hart, Ernest . . . . . . . . . . 541
Hicks, J. Braxton . . . . . . . 230
Hinkley, Alonzo S . . . . . . 698

PAGE.

Hummel, A. L. . . . . . . . . 378
James, Frederick H. . . . . . 58
Johnson, C. C . . . . . . . . 935
Kitchel, Edwin M. . . . . . . . 140
Kollock, Cornelius . . . . 303
Larrabee, John A . . . . . . 934
Lewi, Joseph . . . . . . . . 542
Lewis, George W. . . . . . 231
Lindsley, J. Berrien . . . . 543
Mills, Myron H. . . . . . . . 141
Mooney, Fletcher D . . . . . 379
O'Dwyer, Joseph. . . . . . . 542
Parks, Ravel B. . . . . . . . 379
Parvin, Theophilus . . . . . . . 622
Pean, Jules Emile . . . . . 699
Praeger, E. Arnold . . . . . . . 698
Quain, Sir Richard . . . 699
Quimby, Isaac N. . . . . . . 855
Ransom, Walter J . . . . . . 303
Robb, William H . . . . . . 543
Rodgers, W. O . . . . . . . . 699
Seguin, Edward C . . . . . 624
Sherman, Benjamin F. . . . . 59
Southwick, A. P . . . . . . 935
St. John, Orson S. . . . . . . 58
Storck, Edward. . . . . . . . 138
Talbot, Michael . . . . . . . 623
Tarnier, Prof. . . . . . . . . 543
Tremaine, W. S. . . . . . . 540
Towle, J. J. . . . . . . . 699
Wey, William C. . . . . . . . 54
Wilson, H. P. C. . . . . . . . . 543
Wheaton, Robert A. . . . . . 698
Yandell, David W. . . . . . 854

Ohio Medical Law Sustained . . 284
Registration and Examination Law . . . . . . . . . . . 599
Medical Law, Enforcing the . 607
Ophthalmia Neonatorum . . . . . 25
Treatment of Sympathetic . . . . . . . 432
Gonorrheal, Argonin in . . . . . . . . . 30
How Cost of Might be Lessened . . . . . 585
Orr, C. R., Sputum from Public Places containing Bacillus Tuberculosis . . . . . . . . . . . . 747
Orthoform . . . . . . . . . . . . 683
Osteopathy . . . . . . . . . . . . 848
Oxalic Acid, Action of on Kidney . 429
Oxygen, Tension of Arterial Blood . . . . . . . . . . . . . . . 364

PARALYSIS from Bicycling . . 270
Pediatrics, Opium in . . . . 36
Pennsylvania, New Law in . . . . 280
Perineum, How to Preserve. . . . 689

PAGE.

Peritonitis, Treatment of Tubercular . . . . . . . . 409
Suppurative, New Method of Treatment . . . . 529

**Personal :**

Dunn, B. S.; Hutchinson, Woods; Fronczak, F. E.; Park, Roswell; Wright, A. H.; Durrand, C. F. . . . . . 60
Downer, Louise . . . . . . . 68
Raub, J. F ; Mathews, Jos. M.; Clark, Edward ; Grant, John H.; Sajous, Charles E.; Lewis, Bransford ; Dignen, W. E ; Hartwig, M. . . . . . 137
Weigel, L. A.; Bissell, William G.; Park, Roswell . . . . 230
Wende, Ernest ; Gram, F. C.; Mulford, Henry J.; Hinkel, F. Whitehill ; Smith, Steven; Krauss, William C.; Bacon, John E.; Burroughs, Lorenzo 300
Duff, John Milton ; Suiter, A. Walter ; Reed, R. Harvey ; Park, Roswell ; Spangenthal Joseph. . . . . . . 377
Barrett, Frederic J.; Constantine, D J ; Tremaine, W. S.; Fisher, John C. . . . . 459
Barber, A. H. Freeland ; Benoit, Marie Louise ; Park, Roswell ; Moyer, Harold N.; Sanders W. H. . . . . . . 460
Van Peyma, P. W.; Reed, R. Harvey; Park, Roswell; Hinkel, F. Whitehill; Dignen, W. E. . . . . . . . . 540
Lee, Benjamin; Ross, Jas F W. 619
Brush, Edward N.; House, William ; Williams, Dawson; Lothrop, Thomas ; Hinkel, F. Whitehill ; Hartwig, Marcell . . . . . . . . . . . 697
McLane, James W.; Rolph, Rollin T. . . . . . 783
Renner, W. Scott ; Ruffner, E. L.; Durrand, C. F ; Forsyth, E. A.; Finerty, J. J.; Dowd, J. Henry ; Hanley, L. G.; Palmer, Geo. S. . . . . . . 784
Jones, Allen A. . . . . . . 800
Metcalf, Francis T.; Spangenthal, Joseph ; Mickle, H ; Bayliss, A. W.; Wilson, A. B. Lewis, Daniel . . . . . . 855
Coleman, N. R . . . . . . . 856
House, William . . . . . . . 880
Price, Joseph ; Mathews, Joseph M . . . . . . . . 933
Crego, Floyd S.; Smith, Chauncey Pelton ; Ullman, Julius ; Irving M. Snow . . . . . . 934

Pharmacist as a Milk Dealer . . . 920
Pharyngitis, Follicular . . . . . . 113
Phonendoscope, Use of . . . . . . 531
Physicians as Politicians . . . . . 605
Ex-convicts Cannot be . 848
Placenta Previa . . . . . . . . 175
Two Cases of . . . 423
Plastic Surgery, Interest in . . . 440
Pneumonia . . . . . 844
Poisoning, A Case of Corrosive . . 103
by Pasteurized Milk . . 685
Polyneuritis, Senile. . . . . . . 270
Treatment of . . . . 684
Poor and Needy, Municipal Employment of . . . . . . . . 459
Porro Operation, Successful . . . 437
Post-operative Complications . . . 532
Postpartum Hemorrhage . . . . 690
Potter, William Warren, Puerperal Eclampsia . . . . . . . . . 81
Medical History of the County of Erie . 641, 754, 829
State License to Practise . 669
Practice Law, Enforcement of . . . 539
of Medicine, Constitutionality of Laws Regulating . . . . . . . . . . 604
Preiss, Frederick, Röntgen Ray and Its Usefulness . . . . . . . . . . 807

**Progress in Medical Science :**

Genito-urinary and Syphilitic Diseases, by B. H. Daggett. . 31, 119, 265, 357, 433, 768
Medical Education and State Medical Examinations, by William Warren Potter . . . . . . . . . . 114, 279, 598
Medicine, Pathology and Therapeutics . . . . . . . . . 108, 269, 533, 680, 844, 847
Obstetrics Gynecology and Pelvic Surgery, by William Warren Potter . . . 39, 437, 689
Ophthalmology, by A. A. Hubbell . . . . 24, 354, 430, 688, 765
Pediatrics, by Maud J. Frye. . . . . . . 36, 425, 685, 917
Physiological Chemistry, by John A. Miller . . . . 364, 427
Surgery, by John Parmenter . 529

Pruritus, External Applications for, 629
Vulvæ . . . . . . . . 442
Ani . . . . . . . . . . 610

PAGE.

**Public Health and Hygiene:**

Buffalo the Healthiest City . 465
Garbage Disposal, Death among Plumbers and Formaldehyde Disinfection . . 863
Women's Protective Health Association of Brooklyn 66

Puerperal Eclampsia . . . 39, 81, 440
Fever, Serum Treatment of . . . . . . . . . 40
Diphtheria . . . . . 842
Putnam, James W., Expert Testimony . . . . . . . . . . . . . . 332
Pyothorax, Diagnosis of . . . . . 891
Pyuria, Dr. McMurtry's Discussion on . . . . . . . . . . . . . . 618

QUACKS, Prosecution of in New York . . . . . . . . 288
Quarantine Reform . . . . . 455

REGISTRATION, Compulsory Refused . . . . . . 608
Renal Permeability, Diagnosis of . 124

**Reviews:**

Abbott: Bacteriology . . . . 710
Anders: Practice of Medicine . 472
Annual Report Pennsylvania State Board of Health (1896) . . . . . . . . . 556
Report Supervising-General Marine Hospital Service . . . . . . . . . 154
Ball: Essentials of Bacteriology . . . . . . . . . . 395
Bangs-Hardaway: Genito-urinary Diseases, Syphilis and Skin . . . . . . . . . . . . 869
Bashore: Rural Hygiene . . 709
Beard-Rockwell: Sexual Neurasthenia . . . . . . . . . 875
Bishop: Diseases of the Ear, Nose and Throat . . . . . 151
Boix; Liver of Dyspeptics . 314
Bollinger: Pathological Anatomy . . . . . . . . . . 874
Browning: Circulation in the Nervous System . . . . . . 312
Byford: Manual of Gynecology. 709
Cabot: Clinical Examination of Blood . . . . . . 387
Chapin: Compendium of Insanity . . . . . . 795
Coplin: Manual of Pathology. 794
Crothers: Elements of Latin 715
Currier: The Menopause . . 313

PAGE.

Dana: Nervous Diseases . . 473
Doty: Prompt Aid to the Injured . . . . . . . . . . 942
Duhring: Cutaneous Medicine . . . . . . . . . 466
Fenwick: Disorders of Digestion in Infancy . . . . 236
Fick: Diseases of the Eye and Ophthalmoscopy . . . . . 150
Flint's Medical and Surgical Directory . . . . . . . . . . 152
Foster: Practical Therapeutics . . . . . . . . 70
Fox: Skin Diseases of Children . . . . . . . . . . . 554
Garrigues: Diseases of Women. 632
Gould: American Year-book . 712
Hare: Practical Diagnosis . . 555
Practical Therapeutics . . 553
System of Practical Therapeutics . . . . . . . 237
Hemmeter: Diseases of the Stomach . . . . . . . . . 789
Herold: Legal Medicine . . . 470
Hinsdale: Syringomyelia . . . 636
Holt: Care and Feeding of Children . . . 637
Hopkins: Roller Bandage . 394
Hutchison: Clinical Methods. 710
Index Catalogue Library Surgeon-General's Office . . . 474
International Clinics Vol II., Seventh Series, 313; Vol. III., 468; Vol. IV., 713; Vol. I, Eighth Series . . . . . . 873
Jacobi: Therapeutics of Infancy and Childhood . . . . 874
Jaksch: Clinical Diagnosis . . 469
Jewett: Essentials of Obstetrics . . . . . . . . . . . 474
Johns Hopkins Hospital Reports, Volume VI . . . . 414
Kellogg: Text-book on Mental Diseases . . . 310
King: Manual of Obstetrics . 795
Kirk: Operative Dentistry . . 316
Klemperer: Clinical Diagnosis. 942
Landolt-Gygax: Ophthalmological Therapeutics . 866
Lea Brothers's Year-book of Treatment (1898) . . . . . 714
Lefert: Formulaire . . . . . 874
Lehmann: Essentials of Bacteriology . . . . . . . . . 475
Lilienthal: Surgical Hints . . 154
Lippincott's Medical Dictionary . . . . . . . . . 636
Loomis-Thompson: System of Practical Medicine. Vol. II., 309; Vol. III . . . . . . . 867

PAGE.
Macdonald : Surgical Diagnosis and Treatment . . . . . . 792
Mallory : Pathological Technique . . . . . . . . . . . . 471
Martin : Fibroid Tumors of the Uterus . . . . . . . . . 153
Meigs : Origin of Disease . . 390
Michigan Board of Health, Report . . . . . . . . . 1.4, 796
Mills : Diseases of the Nervous System . . . . . . . . . . . 871
Mitchell : Lessons on Nervous Diseases . . . . . . . . 70
Fat and Blood . . . . . . 941
Monell : Static Electricity in X-ray and Therapeutic Uses. . . . . . . . . . . . 69
Mynter : Appendicitis . . 233
Nettleship : Diseases of the Eye . . . . . . 711
New Hampshire Board of Health Report . . . . . 152
Norris-Oliver : Diseases of the Eye, Volume II . . . . . . 706
Palmer : Inebriety . . . . . 393
Park : History of Medicine . . 555
Penrose : Text-book of Diseases of Women . . . . . 311
Phelps : Traumatic Injuries of the Brain . . . 628
Potter : Materia Medica, etc . 635
Pozzi: Treatise on Gynecology. 552
Preston : Hysteria, etc . . . . 72
Quarantine Stations, Inspections of . . 315
Ranney : Eye-strain in Health and Disease . . . . . . . 148
Report Department of Health, Chicago . . . . . . . 794
State Board of Charities, 1896. . . . . . . . . . 395
of Commissioner of Education, 1895-96. Volume I., Part I., 393 ; Volume II., Part II . . . . . . 791
of Presbyterian Hospital (1897) . . . . . . . . 316
Ringer : Handbook of Therapeutics . . . . . . . . . . 712
Roberts : Fracture of the Radius . . . . . . . . 152
Ruddiman: Incompatibilities in Prescription . . . . . . 633
Sajous : Universal Medical Sciences (1896), 315 ; Analytical Cyclopedia, Vol. I . . . 868
Schaffer : Atlas and Essentials of Gynecology . . 71
Senn : Tuberculosis of Genitourinary Organs . . . . . . 384

PAGE.
Shoemaker : Diseases of the Skin . . . . . . . . . . . . 552
Simon : Clinical Diagnosis . . 407
Sizeranne : Blind Through Blind Eyes . . . . . . . . . 154
Solly : Medical Climatology . 238
Stedman : Twentieth Century Practice. Vol XI., 314 ; Vol. XII., 389 ; Vol XIII . . . . 633
Sutton : Diseases of Women . 391
Taylor: Medical Jurisprudence. 634
Sexual Disorders of the Male and Female . . . 549
Thayer : Malarial Fevers . 472
Transactions : American Association of Obstetricians and Gynecologists, Volume IX . . . . . . . . . 71
American Academy of Railway Surgeons . . . . . 394
American Gynecological Society, Volume XXII . 392
American Medico-psychological Association, 1897. 941
American Microscopical Society (1896), 153 ; (1897) . . . . . . . . . 797
American Surgical Association . . . . . . 714
Association U. S Military Surgeons . . . . . 875
Colorado State Medical Society . . . . . 636
Medical Association of Central New York (1896), . . . . . . . . . 153
Medical Society District of Columbia . . . . . . 942
Medical Society of the State of New York (1897) . . . . . . . . . 236
Medical Society State of North Carolina (1896), 237 ; (1897) . . . . 873
Medical Society State of West Virginia . . 394
National Confederation State Medical Examining Boards . . . 796
Oregon State Medical Society . . . . . . . . 796
Southern Surgical and Gynecological Association, Volume IX . . 151
Texas State Medical Association . . . . . . . . 875
United States Veterinary Medical Association . . 795
Treat's International Medical Annual (1898) . . . . . . . 790

PAGE.

Warner : Pocket Medical Dictionary . . . . . . . . . . 72
Wetterstrand : Hypnotism in Practical Medicine . . . . 392
Whitacre : Text-Book of Pathology . . . . . . . . . 873
White : Genito-urinary Surgery . . . . . . 386
Whiting : Aseptic Technique . 874
Wood : Therapeutics . . . . 635

Rheumatism, Contraindications of Sodium Salicylate in . . . . . . . . . 34
Atypical . . . . . 846
Ringueberg, E. N. S., Quick Test for Visual and Color Fields . . 511
Roe, John O , Unusual Defective Development in an Infant . . . 401
Röntgen Ray and Its Usefulness . 807

SARCOMA, Multiple Idiopathic Pigmented . . . . . . 818
Satterlee, Richard H , New Model of the Javal Ophthalmometer . . 774
Scarlet Fever Patients, Isolation of. 297
Schools, Medical Supervision of . 853
Schröter, L., Three Cases of Hydrocephalus . . . . 259
Science, The Philosophic Spirit in . 321
Sefton, Frederick, Expert Medical Testimony . . . . . . 492
Smith, Eugene A., Diagnosis of Pyothorax . . . . . . . . . . 891
Snow, Sargent F., Adirondacks in Winter, etc . . . . . . . . . . 657

### *Society Meetings :*

American Academy of Medicine . . . . . . 625, 702
American Academy of Railway Surgeons . . . . . 232
American Association of Obstetricians and Gynecologists . 61
American Electro-therapeutic Association . . . . 232, 380, 702
American Medical Association. . . . . . . 702, 787
American Medical Editors' Association . . . . . . . . . 866
American Medical Publishers' Association . 547, 702, 864
American Medical Temperance Association . . . . . . . 864
American Medico-psychological Association . . . . . . 627

PAGE.

American Microscopical Society . . . . . . . . 64, 147
Association Surgeons, Southern Railway Company . . . 701
Buffalo Academy of Medicine. 65, 141, 231, 308, 381, 462, 546, 625, 701, 787 . . . . . 864
Buffalo Sanatory Club . . . . 461
Chicago Pathological Society . 382
Elmira Academy of Medicine . . . . . . . . . 461, 546
Iowa State Medical Society . . 788
Lake Erie Medical Society. . . . . . . . . . 66, 547, 787
Medical Association Central New York . . . . 146, 232, 307
Medical Society County of Cayuga . . . . . . . 864
Medical Society County of Chautauqua . . 65, 380, 461, 936
Medical Society County of Chemung . . . . . . . . . 866
Medical Society County of Erie. . . . . . . 463, 864, 935
Medical Society State of New York . . . . . . . 479, 543, 626
Medico-legal Society of New York . . . . . . . . . 462
Michigan State Board of Health, Quarto-centennial . 788
Mississippi Valley Medical Association . . 66, 144, 307
National Confederation State Medical Examining and Licensing Boards . 627, 701, 865
New York Academy of Medicine . . . . . 382
New York State Association of Railway Surgeons . . . 232, 379
New York State Medical Association, Fourth District Branch . . . . . . . . 865
Niagara Falls Academy of Medicine . . . . . . . . . 627, 700
Ninth International Congress of Hygiene and Demography . . . . . . 547
Ohio State Medical Society . . 786
Physicians' Club of Buffalo. . . . . . . . . . . 148, 863
Roswell Park Medical Club. . . . . . . . . . . . 232, 461
Southern Surgical and Gynecological Association . . 308, 381
St Louis Laryngological and Otological Society . . 547
Thomas J. King Medical Club 936
University State of New York Convocation . . . . . . . . 866

PAGE.

**Society Proceedings:**

American Association of Obstetricians and Gynecologists . 200
Buffalo Academy of Medicine . 528
Medical Society County of Erie. . . . . . 21, 106, 515, 591, 915
Medical Society County of Herkimer . . . . . . . 262
Medical Association of Central New York . . . . . . . . 346
Southern Surgical and Gynecological Association . . . . 594

Specialism, The Prevalence of . . 376
Specialty, My (verses) . . . . . . 854
Spectacles, Vending of . . . . . . 374
Sputum from Public Places Containing Bacillus Tuberculosis . . 747
Stanbro, F. H., Diarrhea in Children . . . . . . . . . . . . . . 581
State Medical Societies and Examining Boards . . . . . . . . . 598
Stephenson, F. H., Jacksonian Epilepsy . . . . . . . . . . . . . 663
Sterility . . . . . . . . . 44
Still-born, New Method of Resuscitating . . . . . . . . . 687
Stomach Tube in General Practice. 896
Strabismus, Operation for . . . . 430
Stricture of the Urethra . . . . . 908
Suiter, A. Walter, The Barber Shop a Menace to Health . . . . . . 801
Supply, Municipal Control of . . . 918
Suprarenal Extracts, Action of . . 429
Surgical Dressing, A New . . . . 131
Syphilis, Thermo-mineral Treatment of . . . . . . . . . . . 266

TEETH, Care of Children's . . 199
Therapeutic Potpourri . . . 273
Thomsen's Disease . . . . . . . 16
Thyroid Gland, Chemistry of . . . 365
Topics of the Time . 295, 373, 454, 538
Tremaine, William Scott, In Memoriam . . . . . . . . . . . 523
Trowbridge, Grosvenor R., Two Cases of Placenta Previa . 423
Tumors of Kidney and Liver, Differentiation of . . . . . . . . . 131
Typhoid Fever . . . . . . . . . 225

PAGE.

ULCERS, Antipyrin in . . . . 534
Ullman, Julius, The Jew in Medicine . . . . . . . . 481
Unsterilised Milk and Death-rate . 917
Umbilic Cord, Picric Acid Dressing. 691
Urea, Formation of by Oxidation . 428
Urethritis, Chronic Specific . . . . 1
Urinary Suppurations . . . . . . 771
Urine, Incontinence of . . . . . . 113
Proteids of Leucemic . . . 365
Excretion of Nitrogen in . 430
Ante-operative Examination of . . . . . . . . . . . 442
Uterus, Objections to Removal of . 41
Lactation Atrophy of . . . 42
Ventrofixation of . . . . . 43
When Shall It be Removed? . . . . . . . . . . . . . 750

VAN FLEET, DR. FRANK, Suit for Damages Against . 295
Vas Deferens, Section of . . . . . 35
Visual and Color Fields, Quick Test for . . . . . . . . . . . . . . . 511
Vitreous Body . . . . . . . . . 689

WATER, Necessity of Drinking . . . . . . . . . . . . 454
Wende, Grover William, Multiple Idiopathic Pigmented Sarcoma . 818
Wende, Dr., and the Schools . . . 933
Wenning, W. H., Treatment of Placenta Previa . . . . . . . . . 175
Whitford, William, Medical Stenographer . . . . . . . . . 458
Whooping Cough, Duration of Infection in . . . . . . . . . . . 50
Williams, Herbert U., Laboratory Methods . . . . . . . . . . . 745
Wisconsin Examining Board . . . 118
Law . . . . . . . . . 282
State Board . . . . . . 847

YELLOW FEVER, Commission to Study . . . . . . . 374
Yellow Fever, Danger of Bringing to United States Mitigated . . . 903

